# Belzutifan efficacy in von Hippel-Lindau disease-associated renal cell carcinoma versus natural history control arm

**DOI:** 10.1093/jnci/djag064

**Published:** 2026-04-08

**Authors:** W Marston Linehan, Thomas Jemielita, Jerry Cornell, Ke Chen, Mark W Ball, Ramaprasad Srinivasan, Yanfang Liu, Rodolfo F Perini, Cathy Anne Pinto

**Affiliations:** Urologic Oncology Branch, National Cancer Institute, Bethesda, MD, United States; Biostatistics and Research Decision Sciences, Merck & Co., Inc, Rahway, NJ, United States; Biostatistics and Research Decision Sciences, Merck & Co., Inc, Rahway, NJ, United States; Biostatistics and Research Decision Sciences, Merck & Co., Inc, Rahway, NJ, United States; Urologic Oncology Branch, National Cancer Institute, Bethesda, MD, United States; Urologic Oncology Branch, National Cancer Institute, Bethesda, MD, United States; Clinical Research, Merck & Co., Inc, Rahway, NJ, United States; Clinical Research, Merck & Co., Inc, Rahway, NJ, United States; Biostatistics and Research Decision Sciences, Merck & Co., Inc, Rahway, NJ, United States

## Abstract

**Background:**

Randomized controlled trials are the gold standard for demonstrating treatment efficacy. When infeasible, external control arm (ECA) analysis is an effective way to interpret treatment arm results. We developed an ECA for a single-arm trial (LS-004) for a HIF-2α inhibitor (belzutifan) in 61 patients with VHL renal cell carcinoma (RCC) to help interpret results. With a median follow-up of 37.8 months in LS-004, the ORR was 64% (95% CI = 50.6 to 75.8); median time to surgery was not reached.

**Methods:**

The ECA was developed using natural history study data for VHL RCC patients undergoing active surveillance with ≤5 years of follow-up. Key LS-004 eligibility criteria were applied. Propensity score (PS) weighting was used to balance prognostic factors, with balance evaluated using standardized mean difference (SMD). PS adjusted point estimates and 95% CI for ORR and time to surgery (TTS) are presented. For the ECA, ORR was evaluated among patients with ≥3 scans to allow opportunity for a confirmed response.

**Results:**

The ECA included 244 patients (167 for ORR analysis). Prognostic factors were balanced with SMD < 0.1 for all covariates. PS adjusted ORR for LS-004 and ECA was 63.9% (95% CI = 51.9 to 76.0) and 1.5% (95% CI = 0.0 to 3.3), respectively. Median TTS was 51.3 (95% CI = 43.8 to not yet reached) months in ECA; not yet reached in LS-004.

**Conclusions:**

The belzutifan treatment effect is large compared with the ECA, supporting belzutifan efficacy in VHL RCC. Although residual confounding is possible, the large effect is unlikely due to chance.

## Introduction

Belzutifan, a first-in-class, selective hypoxia-inducible factor-2 alpha (HIF-2α) inhibitor, is approved for the treatment of von Hippel-Lindau (VHL) disease-associated renal cell carcinoma (RCC), central nervous system (CNS) hemangioblastoma, or pancreatic neuroendocrine tumors (pNET) not requiring immediate surgery.^1^^,^^2^ The approval in VHL patients was based on results of a phase 2 single-arm clinical trial (LITESPARK [LS]-004; NCT03401788) involving 61 patients with VHL disease-associated RCC, CNS HB, and pNET.^1^ Although randomization is the gold standard for evaluating the efficacy of an experimental treatment and minimizing concerns for imbalance of known and unknown prognostic factors with the control, a randomized design is not always possible in certain disease settings. In such cases, natural history studies are increasingly being used to provide indirect and direct comparisons to single-arm therapeutic trials in rare diseases, conditions with unmet medical needs, or where placebo is unethical.^3^^,^^4^ Natural history study data can be used to benchmark treatment efficacy, as separately reported for belzutifan,^5^ and/or can be used to develop an external control arm (ECA) that has similar baseline characteristics as the single-arm trial and provide a comparator for the evaluation of drug effectiveness.^6^ External control arms can be developed using patient-level data from separate ongoing or historical clinical trials or real-world data (RWD) studies.^7-9^

Given the lack of a randomized trial evaluating the use of belzutifan for treating tumors associated with VHL disease, we aimed to strengthen the available evidence of treatment efficacy from the LS-004 single-arm trial to inform the management of VHL RCC. We achieved this goal by studying key efficacy outcomes as observed in the LS-004 trial, specifically focusing on patients who were undergoing active surveillance but did not receive belzutifan therapy or other systemic therapy.

## Materials and methods

The specific objectives of this study were to establish an ECA using RWD from a VHL Natural History (NH) Study and to descriptively compare key efficacy outcomes between LS-004, a phase 2 single-arm registrational trial,^1^ and VHL-RCC NH Study patients.^5^ Differences in key clinical outcomes, including the primary efficacy outcome in the LS-004 trial, were analyzed using propensity scores, which are the propensity for treatment given important baseline characteristics that could affect treatment outcomes. The analysis incorporated inverse probability of treatment weighting (IPTW) as our primary approach and propensity score (PS) matching as a sensitivity analysis.

### Data sources and study population

LS-004 is a single-arm, prospective, interventional phase 2 study which included 61 patients with VHL RCC enrolled at 11 centers in the United States, Denmark, France, and the United Kingdom. The LS-004 data used in the ECA analysis includes a median (range) follow-up of 37.8 (36.1 to 46.1) months. The LS-004 protocol was approved by the appropriate institutional review board or independent ethics committee at each center. All patients in LS-004 provided written informed consent.^1^ The NH Study is a non-interventional, retrospective, observational study that used de-identified clinical data of 244 VHL-RCC patients undergoing active surveillance and receiving comprehensive multidisciplinary clinical and surgical management at the US National Cancer Institute (NCI) and provided informed consent under Protocol 89-C-0086, approved by the NCI Institutional Review Board.^5^ Patients received standard of care clinical management at the National Institutes of Health Clinical Center in Bethesda, Maryland. The NH Study is unique in its alignment with LS-004 design and analysis, including use of an independent review committee (IRC) to review radiographic outcomes per Response Evaluation Criteria in Solid Tumors (RECIST).^10^ NH Study patients met key eligibility criteria defined for the LS-004 single-arm trial (**Table S1**). The patient-level index date was the first date during the study window where patients had a record of RCC measurable disease (≥1 cm solid tumor in longest diameter). For the ECA analysis up to 5 years of follow-up in the NH Study was used.

### Clinical outcomes

The clinical endpoints for the ECA analysis included objective response rate (ORR) and time to surgery (TTS) which is a key secondary endpoint given its clinical relevance. Repeated renal surgeries are common in patients with VHL and are associated with morbidities including CKD and, in some cases, end stage renal failure. ^11^

In LS-004, ORR is defined as the proportion of participants with a best confirmed response of Complete Response (CR) or Partial Response (PR) as determined by RECIST 1.1. RECIST uses the sum of the longest diameters (SLD) of target lesions to assess tumor response to treatment. The analysis of ORR is based on IRC assessments. To closely emulate LS-004, radiographic outcomes in the NH Study were defined using RECIST 1.1 and included disease progression (ie, ≥20% increase in the SLD of target lesions and an absolute increase of ≥5 mm), and objective “response” or regression rate (ORR) including complete regression and partial regression of disease (ie, ≥30% decrease in the SLD of target lesions, with no evidence of disease progression). The radiographic ascertainment window included time from patient-level index date to first of: mortality date or last encounter date; date of receipt of an investigational or oncologic therapy; or first date of a renal procedure impacting any target renal tumor. The primary study population for the RECIST analyses included NH Study patients with a baseline scan at the patient-level index date and ≥2 follow-up scans during the radiographic ascertainment window for target tumor(s) to allow the opportunity for a confirmed response if applicable RECIST criteria were met. Patients were excluded from the ECA ORR analysis if they had <3 serial image assessments (baseline and post-index) as a result of censoring due to surgery within 5 years of follow-up.

For the NH Study and LS-004, TTS was also defined in a similar manner for each study. For LS-004, TTS is defined as the interval from the start of study treatment to the date of first surgery. Participants who do not undergo surgery are censored as of the date of last known alive date. In the NH Study, TTS is defined as the interval from the patient-level index date to the patient’s first renal surgery to reduce the size of the tumor including nephrectomies, ablative procedures, and other VHL-RCC procedures. Participants without any surgery were censored at death or last clinical encounter, whichever occurred first. Radiation therapy is not included in the definition of surgery in each study.

### Analyses

An important first step before development of the ECA was a qualitative assessment of data comparability.^12^ In the absence of randomization, the experimental treatment arm and ECA should be as similar as possible regarding known factors that can affect the outcomes measured. These factors include important baseline characteristics, disease attributes, startup of follow-up for the treatment of interest, concomitant therapies, and clinical outcomes.

Propensity scores were then estimated to develop the ECA for a comparison of LS-004 ORR and TTS outcomes. The primary method of PS adjustment for this analysis was IPTW.^13^ A logistic regression model was used to calculate the propensity of receiving a treatment of interest vs the comparator, denoted as the PS. For patients in the treatment (belzutifan) group, the weight was equal to 1, whereas for patients in the comparator (NH Study) group, the weight was calculated as PS/(1-PS) (the Average Treatment Effect of the Treated [ATT] weights). The weights were stabilized, which entailed dividing the exposure-specific (LS-004 vs NH Study) propensity weights by their respective average.^14^ The ATT IPTW results in a pseudo-population in which NH Study patients with a high probability of receiving treatment have a larger weight and patients with a low probability of receiving treatment have a smaller weight. Thus, the distribution of measured patient characteristics used to calculate the propensity score becomes independent of treatment assignment. PS weighting results in an effective sample size for NH Study patients, which represents the relative sample size after weighting.^15^ Propensity-score adjustment was also performed using 1:1 nearest neighbor matching as a sensitivity analysis. Nearest neighbor matching identifies NH Study participants that are most like the LS-004 participants; all matched NH Study participants subsequently receive a weight of 1 while unmatched NH Study participants receive a weight of 0.

For each approach, balance among baseline prognostic factors was assessed using an absolute standardized mean difference (SMD). Candidate baseline covariates for PS adjustment were selected based on clinical relevance and data availability. An absolute SMD maximum threshold in the range of 0.1 to 0.25 is commonly used to determine good balance among covariates in the study arms.^16^ Using each PS approach, outcomes were analyzed by fitting a weighted logistic regression (for ORR) and weighted Kaplan-Meier (for TTS). For both clinical outcomes, robust sandwich-type standard errors were used to calculate two-sided Wald-based 95% confidence intervals. All analyses were conducted using SAS 9.4 (SAS Institute, Cary NC).

## Results

### Comparability of data from trial and external control arm

A comparison of important factors related to the comparability of LS-004 and ECA and potential for bias is presented in Table 1. This comparison included an assessment of diagnosis, prognosis, treatment and other treatment related effects, geographic region, time periods for data collection, follow-up periods, outcomes, and intercurrent events. Overall, the data are generally comparable for purposes of analysis and suitable for development of the ECA and comparison of clinical outcomes.

**Table 1.** Considerations of comparability of LS-004 single-arm trial and external control arm data generated from natural history study.

Consideration	LS-004 Single-Arm Trial	Natural History (NH) Study	Degree of Potential Bias with External Control Arm (ECA) Analysis
**Diagnosis** Criteria differ based on practice variation/interval between trial and ECA data collected?	Sanger sequencing used for genetic diagnosis	Sanger sequencing used for genetic diagnosis	**Minimal,** as diagnostic techniques are the same
**Prognosis** Similar demographic and clinical characteristics to perform unbiased assessment of the treatment-outcome association?	Important prognostic factors selected based on literature/clinical judgement—age, gender, # tumors, days from surgery to index, von Hippel-Lindau (VHL) deletion, prior RCC surgery	Same variables available in natural history study	**Moderate** Although the same baseline prognostic factors in LS-004 and NH Study are used, there is still a potential for imbalance in missing/unknown prognostic factors given the limited body of research for rare disease
**Treatments** Can ECA data be meaningfully compared to treatment arm data (eg, drug formulation, dose, route of administration)?	Treatment with belzutifan administered per LS-004 protocol	No systemic therapy (patients undergoing active surveillance)	**Minimal,** as there is no active comparator
**Other treatment related factors** eg, prior/concomitant treatments?	Data on prior surgery captured for balance of important prognostic factors Excluded patients with prior systemic therapy use	Data on prior surgery captured for balance of important prognostic factors Excluded patients with prior systemic therapy use	**Minimal,** as data on prior surgery and systemic therapy use are captured
**Geographic region** Standards of care/other factors vary across geographic regions/health care systems?	United States, Western Europe (Denmark, France, Great Britain) participants	United States of America, Canada	**Minimal,** as underlying abnormality in VHL disease (ie, germline VHL gene alterations leading to abnormal HIF-2a stabilization) and surgical management are generally common to all participants
**Time periods** Different time periods for data collection that can impact interpretability of results?	May 31, 2018 (first patient exposed to drug) to present date	July 31, 2004-June 30, 2020 (prior to initial marketing authorization for belzutifan)	**Minimal,** as standard of care and the surgical management approach^a^ has remained essentially constant during the study duration ———- ^a^ active surveillance until the largest renal tumor reaches 3 cm, at which time surgical intervention involving nephron sparing enucleation surgery is recommended
**Follow-up periods** 1. Index date designation consistent?	Follow-up started on day of drug administration	Follow-up started on Patient-Level Index date (first radiographic assessment during study window with measurable RCC [≥10 mm])	**Minimal,** as VHL RCC are generally slow growing tumors with similar eligibility criteria applied with exclusion of tumors >3.0 cm warranting immediate surgery
2. Duration of follow-up periods comparable?	Median duration of follow-up is 3.1 years (range = 0.4–3.8 years). (01APR2022 data cutoff for ECA analysis)	Median duration of follow-up for ECA analysis is up to 5 years	**Minimal,** as follow-up time selected for ECA analysis is comparable to duration of follow-up for LS-004
**Outcomes** 1. Similar definitions?	Objective Response Rate (ORR): Defined per RECIST based on central imaging review and standardized imaging charter Time to Surgery (TTS): Defined as time from start of belzutifan treatment to surgery Surgery is defined as any tumor reducing intervention including partial nephrectomy, radical nephrectomy, ablative procedure (cryoablation, thermal ablation, radioablation, etc), tumor debulking surgeries etc but excluding radiation therapy Participants who do not undergo surgery are censored at last date known alive	ORR: Defined per RECIST based on central imaging review using same imaging lab and similar imaging charter as for LS-004 TTS: Defined as time from patient-level index date to renal surgery Surgery is defined as tumor reducing intervention including nephrectomies, ablative procedures, and other VHL-RCC surgical procedures. It excludes radiation therapy Participants who do not undergo surgery are censored at last date known alive	**Minimal,** as defined in similar manner
2. Differences in data sources?	Data collected prospectively	Data collected retrospectively	**Minimal,** ORR: although there is greater chance for missing/misclassified data in NH Study, minimal information bias is expected given rigor of the radiology assessments in NH Study (RECIST 1.1 by independent committee using same core imaging lab and charter for LS-004) TTS: data on surgery in NH Study is also likely complete given severity of the procedures and, as a result, likely recording of information in the patient’s medical records
3. Timing of outcome assessments?	Frequency of imaging assessments performed on pre-specified schedule	Frequency of imaging assessments performed per routine standard of care	**Moderate,** as less frequent imaging in NH Study may potentially lead to longer observed time to surgery (potential bias not in favor of belzutifan treatment)
4. Similar treatment knowledge?	Investigator not blinded to experimental treatment	Clinician not blinded to lack of treatment	**Moderate,** as time to surgery in LS-004 could be delayed if clinicians expect a delayed treatment effect with the investigational therapy (potential bias in favor of belzutifan treatment).
**Intercurrent events** eg, differential use of additional therapies after treatment initiation	No approved VHL therapy during the study window, with similar censoring criteria for other systemic/locally directed therapies	Active surveillance, with similar censoring criteria for other systemic/locally directed therapies	**Minimal,** as similar censoring criteria are applied

Abbreviations: NH Study = Natural History Study; LS-004 = LITESPARK [LS]-004 (NCT03401788); ECA = external control arm; VHL = von Hippel-Lindau; RCC = renal cell carcinoma, TTS = time to surgery; ORR = objective response rate; RECIST = Response Evaluation Criteria in Solid Tumors; HIF-2α = hypoxia-inducible factor-2 alpha.

### Study population and efficacy outcomes

For PS-weighting, the analysis included 61 patients in the LS-004 trial and 244 patients with VHL-RCC in the NH Study cohort for development of the ECA, among which 178 patients had a baseline scan and ≥2 follow-up scans during the radiographic ascertainment window. For PS-matching, the sample size for the LS-004 trial was reduced from 61 patients to 56 patients as there was no match on baseline prognostic factors for 5 patients in the NH Study.

A summary of key baseline characteristics for LS-004 and NH Study patients in the primary ORR analysis prior to PS weighting is presented in Table 2. In LS-004, the mean age (standard deviation [SD]) at the index date was 41 years (SD = 13.5), and 53% were male. Approximately one-third of participants had a partial or complete VHL gene deletion. The mean size of the largest RCC tumor was 25 mm. Prior to PS weighting, the absolute SMD was ≤0.1 for all covariates, except for the number of baseline RCC target tumors per patient with 1.9 and 2.5 target tumors in the LS-004 and NH Study cohorts, respectively, and the proportion of patients with a renal surgery prior to the index date, with 75% and 65% having a prior renal surgery in the LS-004 and NH Study cohorts, respectively. After PS weighting, there was improved balance among the measured potential prognostic factors in LS-004 and NH Study ECA, with an absolute SMD ≤0.06 for all covariates (Table 2). Similar balance in baseline prognostic factors was observed after PS matching, with an absolute SMD of ≤0.15 for all covariates (**Table S2**).

**Table 2.** Baseline characteristics for LS-004 single-arm trial and natural history study participants, before and after propensity-score weighting.

Variable		LS-004 Single-Arm Trial (*n* = 61)	Natural History Study (*n* = 178)	Observations	Mean Difference	Standard Deviation	Standardized Difference
Age, years	Mean (SD)	41.0 (13.46)	42.3 (12.22)	All	−1.3274	12.857	−0.10324
				PS Weighted	−0.6184		−0.04810
Number of RCC target tumors per patient	Mean (SD)	1.9 (1.1)	2.5 (1.3)	All	−0.6311	1.207	−0.52276
				PS Weighted	−0.0325		−0.02693
Largest RCC baseline tumor per patient, mm	Mean (SD)	25.1 (8.7)	25.1 (10.1)	All	0.0581	9.412	0.00617
				PS Weighted	0.3960		0.04207
Days from last RCC surgery before index date	Mean (SD)	1661.8 (1846.4)	1881.3 (1527.95)	All	37.7417	1642.415	0.02298
				PS Weighted	−33.2274		−0.02023
Male gender	*n* (%)	32 (52.5)	94 (52.8)	All	0.0035	0.499	0.00701
				PS Weighted	−0.0006		−0.00120
Patients with VHL deletion	*n* (%)	18 (29.5)	56 (31.5)	All	−0.0195	0.460	−0.04242
				PS Weighted	0.0031		0.00668
Patients with RCC surgery prior to index date	*n* (%)	46 (75.4)	115 (64.6)	All	−0.1080	0.455	−0.23742
				PS Weighted	−0.0263		−0.05787

All observations are prior to propensity score weighting. Standard deviation of all observations used to compute standardized differences. VHL deletion is partial or complete deletion of VHL gene. Index date is the start of follow-up.

Abbreviations: *n* = total number in LS-004 treatment or Natural History Study cohorts; LS-004 = LITESPARK [LS]-004 (NCT03401788); SD = standard deviation; RCC = renal cell carcinoma; VHL = von Hippel-Lindau; mm = millimeter; PS = propensity score.

**Figure 1.**
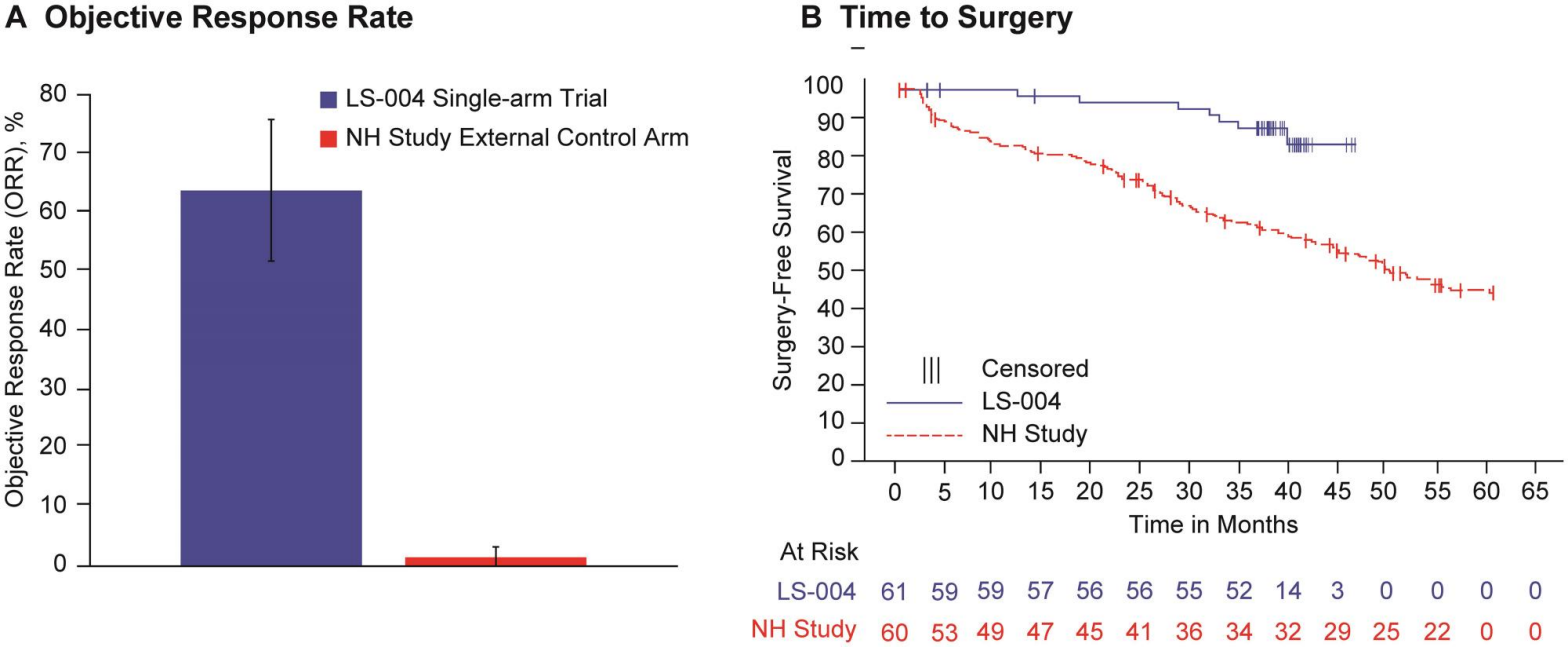
Objective response rate **(A)** and time to surgery **(B)** for LS-004 single-arm trial and natural history study external control arm participants (propensity score weighting). Abbreviations: NH Study = Natural History Study; LS-004 = LITESPARK [LS]-004 (NCT03401788). Date of Data Cut-off: 01APR2022 for LS-004, 05AUG2022 for NH Study. Panel A excludes 11 of the 178 patients from the ORR analysis who had renal surgery and less than 3 serial image assessments (baseline and post patient level index date) required for the RECIST assessment within 5 years of follow-up. Panel B includes an effective sample size of 60 patients in the NH Study after PS weighting.

Using the primary IPTW approach for adjustment, the analysis of surgery included all 61 patients in LS-004 and all 244 patients in the NH Study. The ORR analysis included all 61 patients in LS-004 and 167 of 178 patients with a baseline scan and ≥2 scans during 5 years of follow-up. Eleven patients in the NH Study were excluded from the ORR analysis as a result of censoring due to renal surgery in the 5 year follow-up period. The PS weighted estimate (95% CI) for ORR was 63.9% (95% CI = 51.9 to 76.0) in LS-004 and 1.5% (95% CI = 0.0 to 3.3) for the NH Study ECA (Figure 1,** Panel A**). Among the 167 patients in the NH Study included in the 5-year ORR analysis, the majority (85%) had evidence of disease progression per RECIST 1.1 during follow-up. Seven (11.5%) of 61 participants in LS-004 had surgery compared with 139 (57%) of 244 participants in the NH Study ECA. The median TTS for the NH Study ECA was 51.3 (95% CI = 43.8 to not yet reached) months and not yet reached in LS-004 (Figure 1,** Panel B**). TTS rates at 12 months, 24 months and 36 months were 98.3% (95% CI = 88.6 to 99.8), 84.8% (95% CI = 78.6 to 89.3), and 96.6% (95% CI = 87.0 to 99.1), and 75.6% (95% CI = 68.6 to 81.3), 89.7% (95% CI = 78.5 to 95.2), and 63.5% (95% CI = 55.7 to 70.2) for LS-004 and the NH Study, respectively.

Using the PS-matching approach for adjustment, the analysis of surgery included 56 patients in LS-004 and 56 patients in the NH Study with matched baseline prognostic factors. Four patients were excluded from each study for the ORR analysis as there were 4 patients in the NH Study matched sample who did not have a baseline scan and ≥2 scans during 5 years of follow-up. ORR and TTS results were consistent with the PS-matching approach for adjustment. The PS matched estimate for ORR was 65.4% (95% CI = 50.9 to 70.8) in LS-004 and 3.8% (95% CI = 0.5 to 13.2) in the NH Study ECA. In addition to the improved ORR, the matched analysis showed deeper tumor reduction in LS-004 relative to the NH Study ECA **(Figure S1)**. In the matched analysis, 6 (10.7%) of 56 participants had surgery in LS-004 compared with 34 (60.7%) of 56 participants in the NH Study ECA. The median TTS was 38.2 (95% CI = 26.5 to 59.6) months for the NH Study ECA and not yet reached in LS-004 (**Figure S2**). TTS rates at 12 months, 24 months, and 36 months were 100.0% (95% CI = 100.0 to 100.0), 80.2% (95% CI = 67.1 to 88.5), and 98.1% (95% CI = 87.4 to 99.7), and 67.2% (95% CI = 53.1 to 77.9), 90.6% (95% CI = 78.8 to 96.0), and 51.8% (95% CI = 37.7 to 64.2) for LS-004 and the NH Study, respectively.

## Discussion

Randomized controlled trials are not always feasible, particularly in rare disease settings, where recruitment can be challenging. In addition, there could be questions about the equipoise of randomized studies when there is a significant unmet need alongside promising treatment alternatives and substantial evidence from early-phase clinical trials. Overall, this study offers further evidence supporting the antitumor activity and efficacy of belzutifan in the treatment of VHL-RCC and addresses the evidence gaps in the absence of a randomized controlled trial.

Our analysis employed a measured stepwise approach for development of the ECA for the LS-004 single-arm trial. An important first step was to judge the suitability of developing the ECA and determine if important baseline and clinical characteristics are well characterized, assessed with appropriate methods measured similarly across the groups, and if study analytical methods sufficiently addressed differences in key clinical characteristics between the groups being compared.^12^ Given the unique alignment of the NH Study with the LS-004 single-arm design and analysis, including use of an IRC to review radiographic outcomes using the same imaging lab and imaging charter as for LS-004, the strength of the evidence was deemed suitable for development of the ECA and comparison of key outcomes with LS-004. The most notable differences and potential for bias relate to the assessment of outcomes, specifically the frequency of imaging assessments for LS-004 and NH Study participants. In LS-004, imaging assessments are performed on a pre-specified schedule, whereas in the NH Study, images are obtained in accordance with active surveillance guidelines with much longer periods of time elapsing between imaging assessments in some study participants. Less frequent imaging in the NH Study would lead to longer observed TTS, which would bias the results against the finding of treatment efficacy with belzutifan. With regards to TTS, there could also be a small and likely weaker potential for bias as belzutifan can have a longer time to show an effect, so clinicians may leave patients on the drug longer before making the decision to perform surgery. Even so, the study results were robust with a large and compelling difference in the primary efficacy outcome of response to treatment/regression of disease and TTS, with each assessment demonstrating a large treatment effect with belzutifan. The results of the analyses were consistent, independent of the PS-scoring method applied. The results of this analysis provide further data to support the antitumor effects of belzutifan in VHL disease-associated RCC and are consistent with prior benchmarking data submitted during regulatory agency review of belzutifan to support marketing authorization approval for VHL-RCC based on the LS-004 single-arm registrational trial.^17^^,^^18^

There are additional limitations to this analysis. First, although the ECA analysis was performed according to a prespecified statistical analysis plan, which preceded database lock for analysis, the analysis was performed after results of the NH Study and LS-004 trial were available and were not available at the time of initial regulatory review. Ideally, an ECA analysis should be planned early in the medical product lifecycle at the time of single-arm trial design and initiation of patient enrollment. Upfront planning can further increase transparency and mitigate potential biases in the assessment or interpretation of results. The ECA analysis did not allow for a comparison of other secondary endpoints in LS-004 including time to response, duration of response, and progression-free survival. Duration of response and time to response were not analyzed in the NH Study as patients were followed during active surveillance and there was no active comparator arm nor systemic therapy approved for the treatment of VHL prior to belzutifan. Progression-free survival was an endpoint in the NH Study but not included in the ECA analysis given the high rate of surgery in the NH Study and high degree of censoring in the analysis, potentially biasing the study outcome. Moreover, a comparison of overall survival was also not possible as it was not a study endpoint in LS-004 given the early disease manifestation of VHL and the decades of follow-up and sample size requirements that would be necessary for an OS assessment. Lastly, in terms of potential limitations of this current analysis, although reasonable balance was achieved in known prognostic factors, there are other unknown factors which may not be controlled for in this analysis. The challenge with PS modeling for rare diseases is there is often limited evidentiary basis for selection of prognostic factors. That said, the selection of prognostic factors was based on best available evidence at the time of the analysis and known clinical relevance of these factors.

The magnitude of the treatment effect with belzutifan is large compared with the ECA. These results further support the efficacy of belzutifan relative to observation in patients with VHL disease-associated RCC. Although residual confounding is still possible, such a large effect including a 30-fold greater response rate is unlikely due to chance.

## Supplementary Material

djag064_Supplementary_Data

## Data Availability

The data sharing policy, including restrictions, of Merck Sharp & Dohme LLC, a subsidiary of Merck & Co., Inc., Rahway, NJ, USA (MSD), is available at https://trialstransparency.msdclinicaltrials.com/policies-perspectives.aspx. Requests for access to the clinical study data can be submitted via email to the Data Access mailbox (mailto: dataaccess@msd.com).
