# Supplementary material for: Belzutifan efficacy in von Hippel-Lindau disease-associated renal cell carcinoma versus natural history control arm
Source: J Natl Cancer Inst. 2026 Apr 8;118(6):1081–8. doi: 10.1093/jnci/djag064 (PMC13247334; doi:10.1093/jnci/djag064)
Supplement: djag064_Supplementary_Data [file djag064_supplementary_data.docx]

**Supplementary Material**

**Table S1. Main Eligibility Criteria for LS-004 Single-Arm Trial and Corresponding Criteria Applied for Selection of Natural History Study Participants**

|  | **LS-004 Single Arm Trial Criteria** | **Natural History Study Criteria** |
| --- | --- | --- |
| **Inclusion Criteria** | - resident of Unite States or Western Europe; | - resident of United States of America or Canada; |
|  | - diagnosed von Hippel-Lindau (VHL) disease, based on a germline VHL mutation; | - diagnosed VHL disease and germline mutation; |
|  | - ≥1 measurable solid RCC tumor and no tumor >3.0 cm that requires immediate surgical intervention. | - ≥1 measurable renal solid tumor measured during the study window. |
| **Exclusion Criteria** | Prior to or on the Screening date, | Prior to or on the Participant-Level Index date, |
|  | - received prior treatment with belzutifan or another HIF-2α inhibitor; | - received prior treatment with belzutifan or treatment with HIF-2α inhibitor; |
|  | - received any systemic anti-cancer therapy (includes anti-vascular endothelial growth factor [VEGF] therapy or any systemic investigational anti-cancer agent); | - received systemic anti-cancer therapy (includes anti-VEGF therapy or any systemic investigational anti-cancer agent); |
|  | - had evidence of metastatic disease on screening imaging; | - had evidence of metastatic disease; |
|  | - had radiotherapy or surgical procedure for VHL disease or any major surgical procedure completed within 4 weeks prior to study enrollment, or had an immediate need for surgical intervention for tumor treatment. | - had renal procedures within prior 30 days, or had renal solid tumor ≥3.0 cm at Patient-Level Index Date with a renal surgery performed within 60 days on or after the Participant-level Index date (intended to align with LS-004 criteria of excluding participants who warranted immediate surgery). |

**Table S2. Baseline Characteristics for LS-004 Single-Arm Trial and Natural History Study, Before and After Propensity-Score Matching**

| **Variable** | | **Before Matching** | | **After Matching** | | **Observations** | **Mean Difference** | **Standard Deviation** | **Standardized Difference** |  |
| --- | --- | --- | --- | --- | --- | --- | --- | --- | --- | --- |
|  |  | **LS-004 Single-arm Trial**  **(N=61)** | **Natural History Study**  **(N=178)** | **LS-004 Single-arm Trial**  **(N=56)** | **Natural History Study**  **(N=56)** |  |  |  |  |  |
| **Age, Years** | **Mean (SD)** | 41.0 (13.46) | 42.3 (12.22) | 41.9 (13.1) | 41.0 (12.9) | **All** | -0.8951 | 12.823 | -0.06980 |  |
|  |  |  |  |  |  | **PS Matched** | -0.2786 |  | -0.02173 |  |
| **Number of RCC target tumors per patient** | **Mean (SD)** | 1.9 (1.1) | 2.5 (1.3) | 1.9 (1.1) | 2.0 (1.1) | **All** | -0.5943 | 1.215 | -0.48898 |  |
|  |  |  |  |  |  | **PS Matched** | -0.0179 |  | -0.01469 |  |
| **Largest RCC baseline tumor, mm, per patient** | **Mean (SD)** | 25.1 (8.7) | 25.1 (10.1) | 24.4 (7.4) | 24.9 (10.1) | **All** | -1.3115 | 9.937 | -0.13198 |  |
|  |  |  |  |  |  | **PS Matched** | -1.5000 |  | -0.15095 |  |
| **Days from last RCC surgery before the Index Date** | **Mean (SD)** | 1661.8 (1846.4) | 1881.3 (1527.95) | 1785.1 (1918.5) | 1645.8 (1422.9) | **All** | -1.4836 | 1731.937 | -0.00086 |  |
|  |  |  |  |  |  | **PS Matched** | -92.8750 |  | -0.05362 |  |
| **Male Gender** | **n (%)** | 32 (52.5) | 94 (52.8) | 30 (53.6) | 27 (48.2) | **All** | 0.0205 | 0.499 | 0.04109 |  |
|  |  |  |  |  |  | **PS Matched** | -0.0357 |  | -0.07162 |  |
| **Patients with VHL Deletion** | **n (%)** | 18 (29.5) | 56 (31.5) | 15 (26.8) | 18 (32.1) | **All** | 0.0041 | 0.455 | 0.00900 |  |
|  |  |  |  |  |  | **PS Matched** | -0.0536 |  | -0.11770 |  |
| **Patients with RCC Surgery Prior to Index Date** | **n (%)** | 46 (75.4) | 115 (64.6) | 41 (73.2) | 40 (71.4) | **All** | -0.1025 | 0.454 | -0.22562 |  |
|  |  |  |  |  |  | **PS Matched** | 0.0000 |  | 0.00000 |  |
| N= total number in LS-004 treatment or Natural History Study cohorts. LS-004= LITESPARK [LS]-004 (NCT03401788); SD= standard deviation; RCC= renal cell carcinoma; VHL=von Hippel-Lindau; PS=propensity score. All observations are prior to propensity score weighting. Standard deviation of All observations used to compute standardized differences. VHL deletion is partial or complete deletion of VHL gene. Index date is the start of follow-up. | | | | | | | | | | |

**Figure S1. Maximum Percent Reduction in Total Sum of RCC Target Lesions Diameters from Baseline to Post-Baseline for LS-004 Single-arm Trial (Panel A) and Natural History Study Participants (Panel B) by Best Confirmed Overall Response (Propensity-Score Matching)**


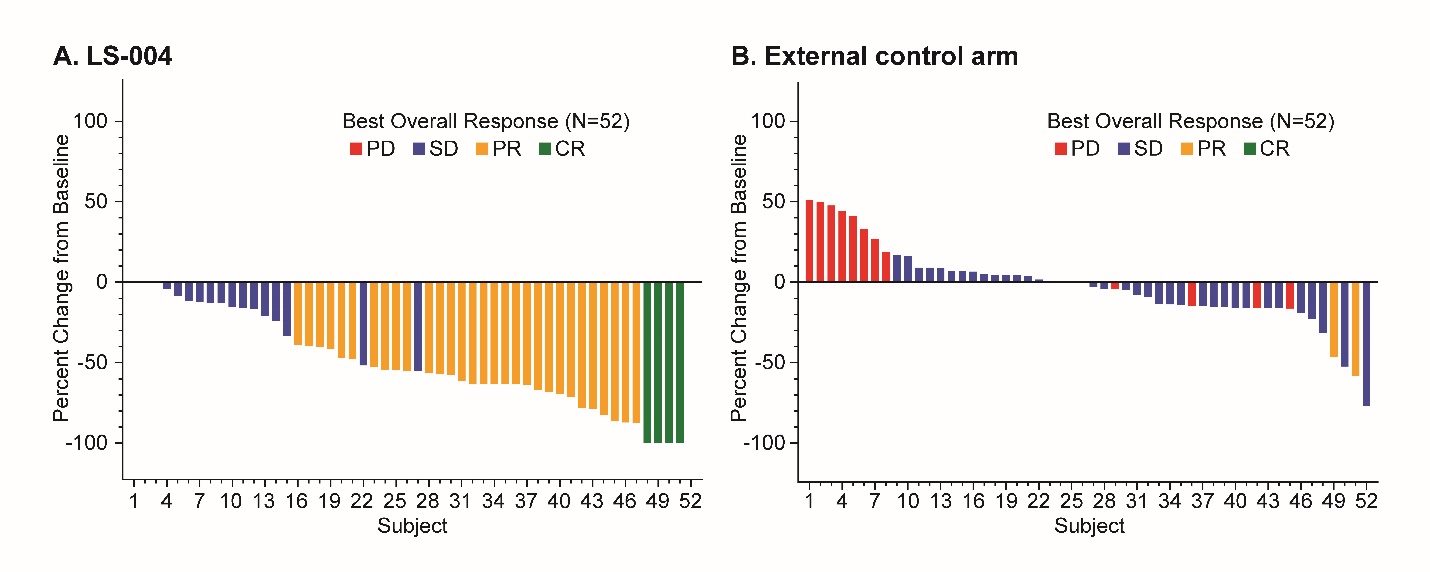


Abbreviations: NH Study=Natural History Study; LS-004=LITESPARK [LS]-004 (NCT03401788); PD=progressive disease, SD=stable disease, PR=partial response, CR=complete response according to RECIST 1.1 criteria. Percent change from baseline represents maximum reduction in sum of longest diameters of baseline target tumors. Panels A and B exclude 4 of 56 patients from the matched analysis as 4 patients in the NH Study had renal surgery and less than 3 serial image assessments (baseline and post patient level index date) required for the RECIST assessment within 5 years of follow-up. Panel B includes 4 patients with best overall response of PD with a maximum percent reduction <0 prior to disease progression; 90% of patients had evidence of disease progression per RECIST 1.1 during follow-up.

**Figure S2. Kaplan-Meier Plot of Time to Surgery for LS-004 Single-Arm Trial and Natural History Study Patients (Propensity-Score Matching)**


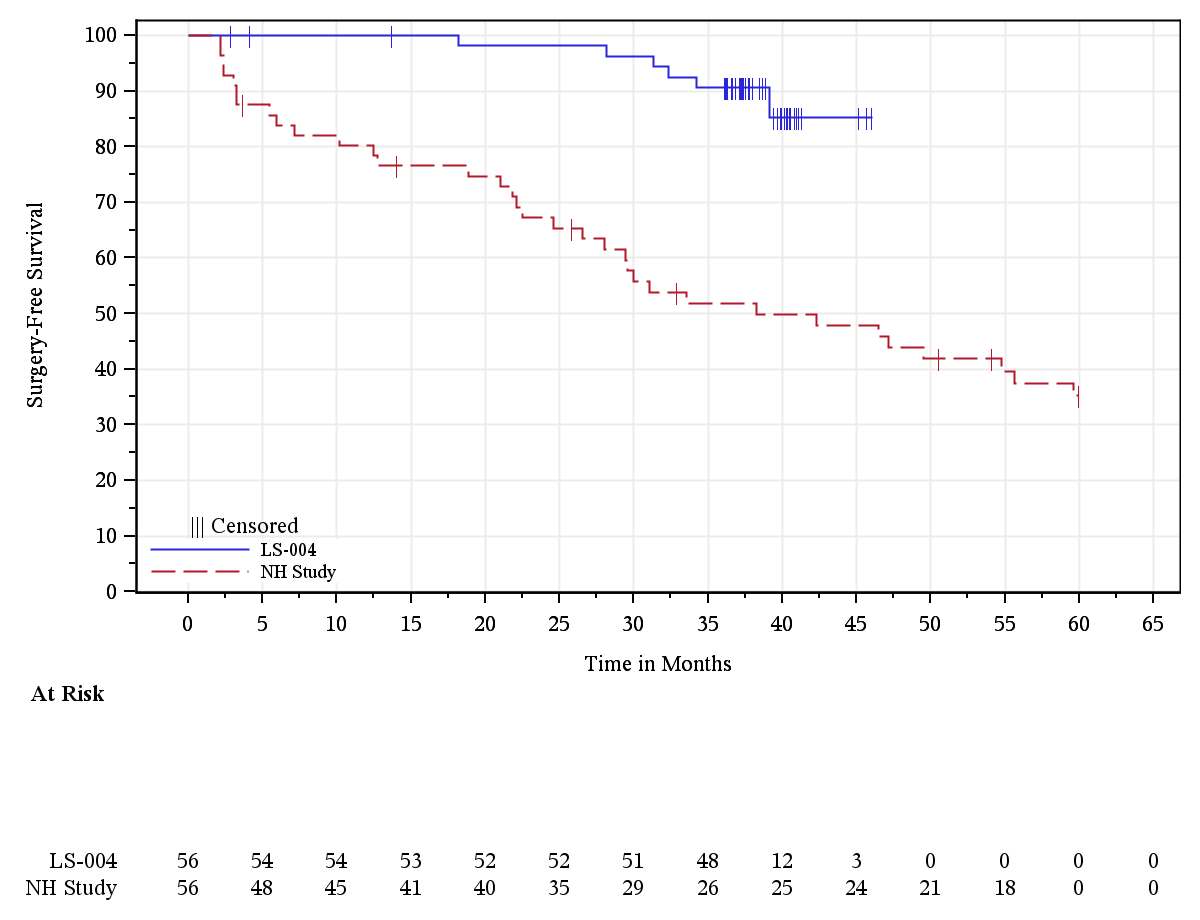


Abbreviations: NH Study=Natural History Study; LS-004=LITESPARK [LS]-004 (NCT03401788)

Date of Data Cut-off: 01APR2022 for LS-004, 05AUG2022 for NH Study. The analysis includes 56 patients in LS-004 trial and 56 patients in NH Study matched on baseline prognostic factors.
